# Psychometric evaluation of EQ-5D-5L in OHCA survivors from the TTM2 trial: a *post hoc* analysis

**DOI:** 10.1016/j.resplu.2025.100994

**Published:** 2025-05-29

**Authors:** Mattias Bohm, Kristofer Årestedt, Susann Ullén, Niklas Nielsen, Josef Dankiewicz, Hans Friberg, Erik Blennow Nordström, Alain Cariou, Janus Christian Jakobsen, Anders Morten Grejs, Matthias Haenggi, Naomi E. Hammond, Katarina Heimburg, Thomas R. Keeble, Christoph Leithner, Christian Rylander, Johan Undén, Matt P. Wise, Tobias Cronberg, Gisela Lilja

**Affiliations:** a Brain Injury After Cardiac Arrest Research Unit, Department of Clinical Sciences Lund, Lund University, Lund, Sweden; b Department of Intensive and Perioperative Care, Skåne University Hospital, Malmö, Sweden; c Faculty of Health and Life Sciences, Linnaeus University, Kalmar, Sweden; d Department of Research, Region Kalmar County, Kalmar, Sweden; e Clinical Studies Sweden – Forum South, Skåne University Hospital, Lund, Sweden; f Department of Clinical Sciences Lund, Lund University, Lund, Sweden; g Department of Anesthesiology and Intensive Care, Helsingborg Hospital, Helsingborg, Sweden; h Department of Cardiology, Skåne University Hospital, Lund, Sweden; i Department of Rehabilitation Medicine, Skåne University Hospital, Lund, Sweden; j Medical Intensive Care Unit, Cochin University Hospital, AP-HP Centre Université Paris Cité, Paris, France; k Copenhagen Trial Unit, Centre for Clinical Intervention Research, Capital Region of Denmark, Denmark; l Department of Regional Health Research, The Faculty of Health Sciences, University of Southern Denmark, Denmark; m Department of Intensive Care Medicine, Aarhus University Hospital, Denmark; n Department of Clinical Medicine, Aarhus University, Aarhus, Denmark; o Institute of Intensive Care Medicine, University Hospital Zurich, Zurich, Switzerland; p Critical Care Program, The George Institute for Global Health, UNSW Sydney, Barangaroo, Australia; q Malcolm Fisher Department of Intensive Care, Royal North Shore Hospital, Northern Sydney Local Health District, St Leonards, Australia; r Essex Cardiothoracic Centre, Nethermayne, Basildon, Essex, UK; s ARU School of Medicine & MTRC, Chelmsford, Essex, UK; t Charité – Universitätsmedizin Berlin, corporate member of Freie Universität Berlin and Humboldt-Universität zu Berlin, Department of Neurology and Experimental Neurology, Augustenburger Platz 1, 13353 Berlin, Germany; u Anesthesiology and Intensive Care, Department of Surgical Sciences, Uppsala University, Uppsala, Sweden; v Anaesthesiology and Intensive Care Medicine, Department of Clinical Sciences Malmö, Lund University, Malmö, Sweden; w Department of Operation and Intensive Care, Hallands Hospital, Halmstad, Sweden; x Adult Critical Care, University Hospital of Wales, Cardiff, UK; y Department of Neurology, Skåne University Hospital, Lund, Sweden

**Keywords:** Heart arrest, Health status, Patient reported outcome measures, Psychometrics, Validation study, Cross-sectional studies

## Abstract

**Aims:**

Our aim was to investigate the psychometric properties of the health assessment instrument EQ-5D-5L in OHCA survivors.

**Methods:**

We included survivors from the Targeted Hypothermia versus Targeted Normothermia after OHCA (TTM2) trial, who completed EQ-5D-5L at 6 months. Confirmatory factor analysis was used to evaluate the hypothesised unidimensional latent structure of EQ level sum score (EQ LSS), summarizing scores across *Mobility*, *Self-care*, *Usual activities*, *Pain/discomfort*, and *Anxiety/depression*. Differential item functioning was evaluated for age. We examined internal consistency and precision for the EQ LSS. We evaluated construct validity of EQ LSS, EQ value and EQ VAS, using the modified Rankin Scale and Montreal Cognitive Assessment, representing functional outcome and cognitive function—two common health challenges experienced by OHCA survivors.

**Results:**

783 of 939 (84%) eligible survivors were included. Confirmatory factor analysis showed good model fit and strong factor loadings for all dimensions (0.61–0.90). We observed a significant but negligible effect of age on Mobility (β = 0.29, p < 0.001, ΔR^2^ = 0.019). Internal consistency was 0.88. The floor effect was 35%. Survivors with more functional dependency and/or cognitive problems reported significantly worse health by EQ LSS, EQ value, and EQ VAS (all, p < 0.001).

**Conclusion:**

The psychometric properties of EQ LSS support its use to measure health status in OHCA research. The strong association between health and functional dependency indicate robust and comparable construct validity for EQ LSS, EQ value, and EQ VAS in this sample.

## Introduction

Survivors of out-of-hospital cardiac arrest (OHCA) often face significant health challenges.^1^, ^2^ EQ-5D-5L, a widely used generic instrument for assessing health status,^3^ is included as a recommended assessment in the Core Outcome Set after Cardiac Arrest (COSCA),^1^ but its psychometric properties have not been evaluated in OHCA survivors.

A common approach to compare health across groups using EQ-5D-5L is with the EQ value (also called EQ index), calculated from population values sets.^3^, ^4^ EQ value was developed for health-economic evaluations but often serves as a shortcut to facilitate statistical analyses in research.^3^, ^4^ It has been criticized for not adequately capturing individual experiences and increasing the risk of statistical bias.^4^

Alternatively, health status can be presented with the EQ level sum score (EQ LSS), summarizing all dimensions as a single value. It may reflect an individual’s health better than the EQ value because it does not rely on societal values.^5^ The straightforward summation makes it practical for research settings. Recently, the EQ LSS was shown to be a scalable health measure.^6^ Support for the unidimensional latent construct of EQ LSS using confirmatory factor analysis (CFA) has previously been reported,^7^, ^8^ while others suggest a more complex structure.^9^, ^10^ To justify the use of EQ LSS scale in OHCA research, the latent structure need to be evaluated in this population.

The EQ VAS is an additional way to present health by EQ-5D-5L, reflecting the respondent’s perceived overall health as a single score.^11^ It is a separate part of the EQ-5D-5L and is not included in the calculations of EQ value or EQ LSS.

Another important psychometric property is the absence of differential item functioning (DIF), meaning items perform differently across subgroups.^12^ Age is a key factor in DIF analyses.^13^ It is unknown whether the EQ-5D-5L exhibits age-related DIF amongst OHCA survivors. The aim of this study was to investigate psychometric properties of the EQ-5D-5L in OHCA survivors and more specifically, to evaluate:

I) The hypothesised unidimensional latent structure of EQ LSS.
II) DIF for age.
III) Internal consistency and precision for EQ LSS.
IV) Construct validity of EQ LSS, EQ value and EQ VAS. We hypothesised that survivors with more functional dependency and cognitive problems report more health problems.

## Methods

### Study design

We use data collected in the Targeted Hypothermia versus Targeted Normothermia after Out-of-Hospital Cardiac Arrest (TTM2) trial.^14^ The TTM2-trial compared hypothermia at 33 °C versus normothermia at ≤ 37.5 °C after OHCA. Rationale and design of the TTM2-trial and the protocol for outcome reporting have been published previously.^15^, ^16^

### Sample

The TTM2-trial, conducted at 61 sites in 14 countries, enrolled adult (18 years or older) unconscious patients after OHCA between November 2017 and January 2020. The sample in this study consists of survivors who participated in the 6-month follow-up with self-completed responses on the EQ-5D-5L questionnaire.

### Data collection

EQ-5D-5L was collected during the follow-up, mainly performed at an institution or, if not possible, at the participant’s residence or by telephone. It was completed as the first part of the follow-up to minimize influence of other assessments. Outcome assessors could read the items and response categories verbatim, but were not allowed to discuss the answers.^16^

### Primary outcome measure

The generic health questionnaire EQ-5D-5L comprises five dimensions: *Mobility, Self-care, Usual activities, Pain/discomfort, and Anxiety/depression*. Each dimension has five ordinal response categories, from no problems (1) to extreme problems (5).^11^, ^17^ EQ LSS summarizes the responses across the five dimensions, ranging from 5 (no problems in any dimension) to 25 (extreme problems in all dimensions).

The five dimensions can be combined to present 3125 (5^5^) different health state profiles, from 11,111 (no problems in any dimension) to 55,555 (extreme problems in all dimensions). A EQ value can be calculated using national value sets derived from a general public evaluating different health states,^5^, ^11^ typically ranging from < 0 (a health status considered worse than death) to 1 (best possible health status). The Swedish EQ value set,^18^ was used to calculate the EQ value for all survivors in this study, as Sweden had most respondents with complete EQ-5D-5L data.

EQ VAS is a vertical visual analogue scale, ranging from 0 (worst imaginable health) to 100 (best imaginable health).^11^

### Additional outcome measures

Functional outcome, cognitive function, and life satisfaction were used to assess the construct validity of EQ-5D-5L.

Functional outcome was assessed with modified Rankin Scale (mRS), an ordinal scale from 0 (no symptoms) to 6 (death),^19^ included in the COSCA.^1^ The assessment was based on the standardised nine questions interview.^20^

Cognitive function was assessed with the performance-based Montreal Cognitive Assessment (MoCA),^21^ which performs well for cognitive screening after cardiac arrest.^22^, ^23^ The range is 0–30 points; higher scores indicate better cognitive performance, and scores < 26 indicate cognitive problems.^24^

Life satisfaction was assessed with a single-item question: *“All things considered, how satisfied are you with your life as a whole these days?”*,^25^ reported on an ordinal scale ranging from 1 (completely dissatisfied) to 10 (completely satisfied).

### Sociodemographic and clinical variables

Prehospital, sociodemographic, and clinical variables obtained at the follow-up or from the TTM2-trial database, are described in Table 1.

**Table 1:** Characteristics of the OHCA survivors from the TTM2-trial participating in the 6-month follow-up with complete responses on EQ-5D-5L (n = 783).

**Pre-arrest variables**	
Age [years], mean (SD)	59.9 (13.5)
Age groups, n (%)	
18–65 years	493 (63)
66–75 years	211 (27)
>75 years	79 (10)
Male sex, n (%)	657 (84)
Charlson comorbidity index (CCI) [0–33], Mdn (Q1–Q3) a	2 (1–3)
Comorbidity groups based on CCI score, n (%) b	
0 points	131 (17)
1 point	151 (19)
2 points	178 (23)
3 points	146 (19)
≥4 points	177 (23)
Highest formal education level, n (%) c	
No formal education	4 (< 1)
Primary/lower secondary school	211 (27)
Upper secondary school	302 (39)
University-level education, with or without degree	260 (33)
Married or living as married [yes], n (%)	583 (75)
**Cardiac arrest related variables**	
ROSC [minutes], Mdn (Q1–Q3)	20 (14–30)
Bystander CPR [yes], n (%)	671 (86)
LOS ICU [days], Mdn (Q1–Q3)	5 (3–9)
LOS hospital [days], Mdn (Q1–Q3)	15 (10–24)
**Status at 6-month follow-up**	
modified Rankin Scale (mRS), n (%)	
0 [No symptoms]	281 (36)
1 [No significant disability]	164 (21)
2 [Slight disabilities]	250 (32)
3 [Moderate disabilities]	58 (7)
4 [Moderately severe disability]	28 (4)
5 [Severe disability]	2 (0.2)
MoCA score [0–30], Mdn (Q1–Q3)	27 (24–29) [n = 741]
Cognitive outcome groups, n (%)	
No problems [MoCA ≥ 26]	449 (61)
Problems [MoCA < 26]	292 (39)
Living at home [yes], n (%)	762 (98)
Life satisfaction [1–10], Mdn (Q1–Q3) d	8 (7–9)

Abbreviations: CPR – Cardiopulmonary resuscitation; ICU – Intensive care unit; LOS – Length of stay; Mdn – median; OHCA – out-of-hospital cardiac arrest; Q1–Q3 – quartile 1 to 3; ROSC – Return of spontaneous circulation; SD – Standard deviation; TTM2-trial – Targeted Hypothermia versus Targeted Normothermia after Out-of-Hospital Cardiac Arrest trial.

a Charlson Comorbidity Index was used to indicate pre-arrest comorbidity burden, with 0 points indicating no recorded or known pre-arrest comorbidity and higher scores indicate greater burden.

b Respondents were categorized based on their Charlson Comorbidity Index score.

c Education level was based on the International Standard Classification of Education. Based on 777 observations.

d Higher values indicate better overall life satisfaction. Based on 778 observations.

### Statistical analyses

Descriptive statistics are reported as numbers and percentages, mean and standard deviation, or median and quartile 1–3. When appropriate, a confidence interval (CI) is reported. This study was guided by reporting guidelines from the Consensus-based standards for the selection of health measurement instruments (COSMIN) initiative with its terminology adapted accordingly.^26^, ^27^ Table S1 presents detailed information on the different methods used to evaluate psychometric properties, including statistical analyses, criteria, and interpretation.

I) *Latent structure*

A CFA for ordinal indicators was used to evaluate the hypothesised unidimensional latent structure of EQ LSS. The CFA was based on polychoric correlations with weighted least square mean and variance (WLSMV) adjusted estimation. Goodness-of-fit was estimated with standard indices (Table S1).^28^ Standardized factor loading were expected ≥ 0.5.^29^

II) *Differential item functioning for age*

DIF was assessed using a *multiple indicators, multiple causes (MIMIC)* CFA model. A baseline model regressing the latent factor on age (continuous variable), was compared with five separate extended nested models to test for item-specific DIF. The models were compared using scaling corrected χ^2^-difference test,^30^ with Δ*R*^2^ as effect size.^31^.

III) *Internal consistency and precision*

Internal consistency of the EQ LSS was assessed with ordinal alpha (α) and ordinal omega (ω).^32^

Score precision of the EQ LSS was estimated with standard error of measurement (SEM).^33^

IV) *Construct validity*

We formulated *a priori* hypotheses for construct validity in accordance with COSMIN guidelines.^26^ Construct validity of EQ LSS, EQ value, and EQ VAS was assessed using the mRS (functional outcome) and MoCA (cognitive function), reflecting common health challenges in OHCA survivors. We hypothesized that survivors with higher mRS scores and/or cognitive problems (MoCA < 26) would report significantly more health problems, with at least small effect sizes expected.^34^, ^35^ The Kruskal-Wallis test (with eta squared $\eta_{H}^{2}:$ < 0.06 small; 0.06–0.14 moderate; > 0.14 large)^36^ was used for mRS, and the Mann-Whitney *U* test (with rank-biserial correlation *r*: < 0.3 small; 0.3–0.5 moderate; > 0.5 large)^37^ for MoCA.

Convergent and discriminant validity, as aspects of construct validity, were assessed with Spearman correlations (ρ). To support convergent validity, strong correlations corresponding to a high effect size (ρ ≥ 0.5) were expected between EQ LSS, EQ value, and EQ VAS as they measure the similar construct. To support discriminant validity, correlations between these measures and the life satisfaction item were expected to be lower as they reflect different constructs.

As a sensitivity analysis, UK and French value sets were used to assess the impact of country-specific value sets on the construct validity of EQ values.^38^, ^39^

All statistical analyses were performed using R Statistical Software,^40^ including the packages: lavaan,^41^ semTools,^42^ and psych.^43^ Statistical significance was set at p < 0.05.

## Results

At 6 months, 817 of 939 (87%) survivors responded on the EQ-5D-5L. Two respondents with missing EQ-5D-5L item data and 32 participants (3%) with proxy ratings were excluded. Participant characteristics of the 783 respondents with complete EQ-5D-5L data are presented in Table 1. Information of respondents per country is presented in Table S2.

Descriptive statistics of EQ-5D-5L are presented in Table 2, with additional information of response frequency of individual items in Table S3. The floor and ceiling effects of EQ LSS was 35% and < 1%, respectively (Table S4).

I) Latent structure of EQ LSS

**Table 2:** Descriptive statistics of health status by the three different EQ-5D-5L scoring methods amongst OHCA survivors from the TTM2-trial participating in the 6-month follow-up (n = 783).

**Variable**	Dispersion statistics
**EQ LSS** [5–25] a	
Mdn (Q1–Q3)	6.0 (5.0–8.0)
Mean (SD)	7.3 (2.9)
**EQ value** [based on Swedish value set: 0.243–0.976] b	
Mdn (Q1–Q3)	0.94 (0.86–0.98)
Mean (SD)	0.90 (0.11)
**EQ VAS** [0–100] c	
Mdn (Q1–Q3)	80.0 (67.0–90.0)
Mean (SD)	75.9 (18.7)

Abbreviations: Mdn – median; OHCA – Out-of-Hospital Cardiac Arrest; Q1–Q3 – quartile 1–3; SD – standard deviation; TTM2-trial – Targeted Hypothermia versus Targeted Normothermia after Out-of-Hospital Cardiac Arrest trial.

a EQ-5D-5L level sum score (EQ LSS), summarizes the responses across the five dimensions, ranging from 5 (no problems in any dimension) to 25 (extreme problems in all dimensions). Higher values indicate worse health. Mean (SD) is reported for comparative reasons.

b EQ value was based on the Swedish value set (Burström K, et al., 2020), ranging from 0.243 to 0.976. Higher values indicate better health. Mean (SD) EQ value in Swedish population norm: 0.9 (0.2).

c EQ VAS was based on 781 observations. It is reported on visual analogue scale, ranging from 0 to 100 (best imaginable health). Higher values indicate better overall health. Mean (SD) EQ VAS in Swedish population norm: 76.1 (18.7).

**Fig. 1:**
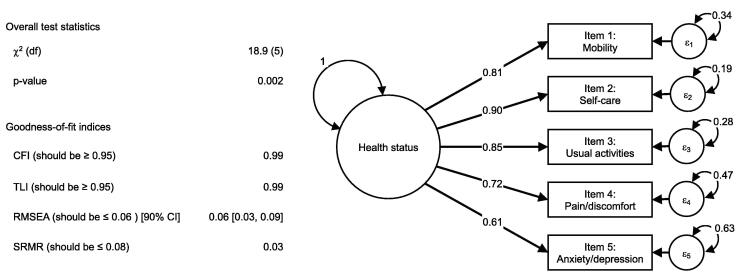
The figure illustrates the confirmatory factor analysis (CFA) of EQ-5D-5L amongst OHCA survivors from the TTM2-trial (n = 783). The CFA was based on polychoric correlations with Weighted Least Square Mean and Variance (WLSMV) adjusted estimation. The model represents an all-effect model with standardised factor loadings and error variances (ε_1–5_). Abbreviations: CFI – Comparative Fit Index; CI – confidence interval; df – degrees of freedom; OHCA – out-of-hospital cardiac arrest; RMSEA – Root Mean Square Error of Approximation; SRMR – Standardized Root Mean Square Residual; TLI – Tucker-Lewis Index; TTM2-trial – the Targeted Hypothermia versus Targeted Normothermia after Out-of-Hospital Cardiac Arrest trial.

The CFA exhibited good model fit and all items demonstrated standardized factor loadings > 0.5 (range: 0.61–0.90), with *Self-care* showing the strongest association with the latent variable and *Anxiety/depression* the weakest (Fig. 1).

II) Differential item functioning for age

DIF for age was detected in three items, where older survivors reported significantly higher scores on *Mobility* (β = 0.29, p < 0.001, Δ*R^2^* = 0.019), and lower scores on *Usual activities* (β = −0.07, p = 0.044, Δ*R^2^* < 0.001) and *Anxiety/depression* (β = −0.24, p < 0.001, Δ*R^2^* = 0.017) than expected based on the total score (Table S5).

III) Internal consistency and precision

The internal consistency of EQ LSS was 0.88 (ordinal alpha) and 0.81 (ordinal omega). The SEM was 1.01 (Table S4).

IV) Construct validity

**Fig. 2:**
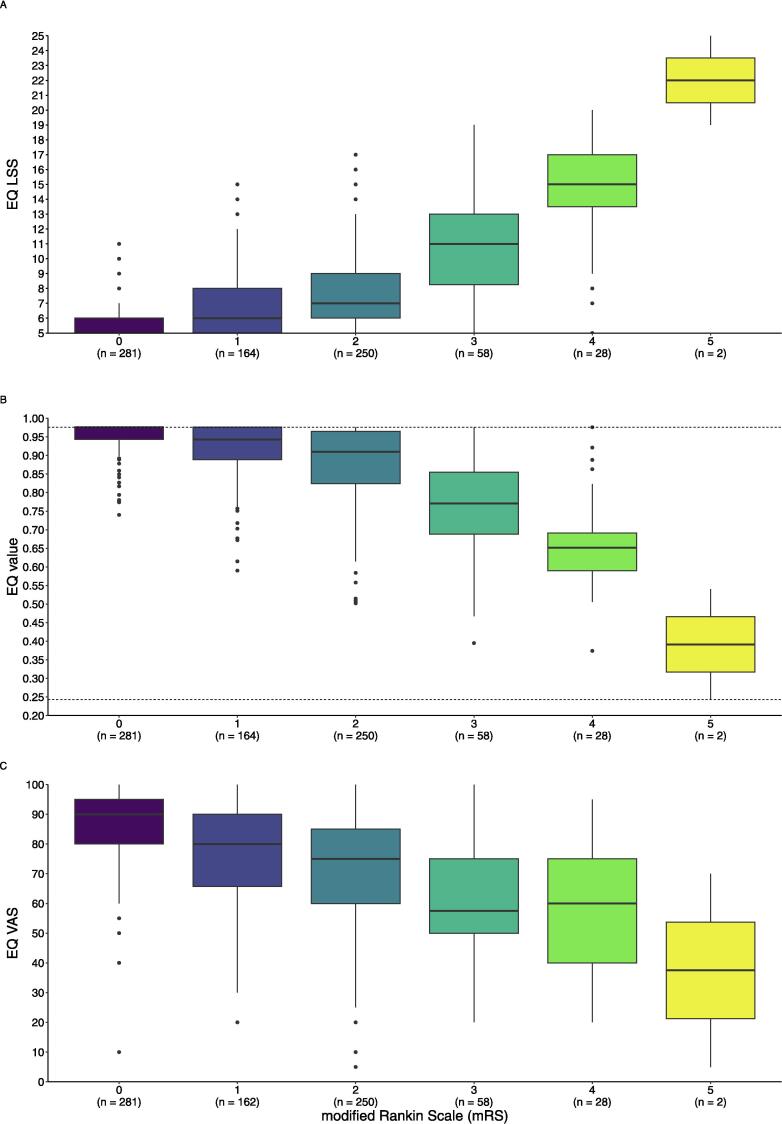
Illustration of the association between health by EQ-5D-5L and modified Rankin Scale (mRS) categories (n = 783). A. EQ level sum score (EQ LSS) ranges between 5 and 25, with higher values indicate more health problems. B. EQ value was based on the Swedish value set, which ranges between 0.243 and 0.976 (indicated by the horizontal dashed lines), with higher values indicating less health problems. C. EQ VAS ranges between 0 and 100, with higher values indicating better overall health.

As hypothesized, survivors with greater functional dependency (mRS) reported significantly more health problems, with large effect sizes: EQ LSS and EQ value (both, $\eta_{H}^{2}$ = 0.35, p < 0.001), and EQ VAS ($\eta_{H}^{2}$ = 0.18, p < 0.001) (Table 3). Fig. 2 illustrates the association between health and mRS categories. There were significant differences between those with and without cognitive impairment for EQ LSS, EQ value, and EQ VAS (all, p < 0.001), but with small effect sizes (*r* = 0.15–0.20) (Table 3).

**Table 3:** Construct validity of EQ-5D-5L, amongst OHCA survivors at 6 months from the TTM2-trial (n = 783).

Property	EQ LSS	EQ value [Swedish value set]	EQ VAS
**Hypotheses testing**			
modified Rankin Scale (mRS)			
0 [n = 281], Mdn (Q1–Q3)	5 (5–6)	0.98 (0.94–0.98)	90 (80–95)
1 [n = 164], Mdn (Q1–Q3)	6 (5–8)	0.94 (0.89–0.98)	80 (66–90) [n = 162]
2 [n = 250], Mdn (Q1–Q3)	7 (6–9)	0.91 (0.82–0.97)	75 (60–85)
3 [n = 58], Mdn (Q1–Q3)	11 (8–13)	0.77 (0.69–0.86)	58 (50–75)
4 [n = 28], Mdn (Q1–Q3)	15 (14–17)	0.65 (0.59–0.69)	60 (40–75)
5 [n = 2], Mdn (Q1–Q3)	22 (21–24)	0.39 (0.32–0.47)	38 (21–54)
p-value a	< 0.001	< 0.001	< 0.001
Effect size ($\eta_{H}^{2}$) b	0.35	0.35	0.18
Montreal Cognitive Assessment (MoCA)			
No cognitive problems [MoCA ≥ 26], Mdn (Q1–Q3)	6 (5–7)	0.95 (0.90–0.98)	80 (70–90)
Cognitive problems [MoCA < 26], Mdn (Q1–Q3)	7 (5–10)	0.92 (0.79–0.98)	75 (60–90)
p-value c	< 0.001	< 0.001	< 0.001
Effect size (*r*) d	0.20	0.20	0.15
**Convergent validity**^e^			
EQ value [Swedish value set] f	–0.99 [–0.99, –0.98]		
EQ VAS	–0.59 [–0.63, –0.54]	0.59 [0.54, 0.63]	
**Discriminant validity**^e^			
Life satisfaction	–0.50 [–0.55, –0.44]	0.51 [0.46, 0.56]	0.58 [0.53, 0.63]

Abbreviations: EQ LSS – EQ-5D-5L level sum score; Mdn – median; OHCA – out-of-hospital cardiac arrest; TTM2-trial – Targeted Hypothermia versus Targeted Normothermia after Out-of-Hospital Cardiac Arrest trial.

a Kruskal-Wallis test.

b Eta squared ($\eta_{H}^{2}$) effect size: < 0.06 (small effect), 0.06–0.14 (moderate effect), and > 0.14 (large effect).

c Mann-Whitney *U* test.

d Rank-biserial correlation coefficient (r) effect size: < 0.3 (small effect), 0.3–0.5 (moderate effect), and > 0.5 (large effect).

e Spearman’s rho. Values in square brackets indicate the 95% confidence interval. A coefficient of ρ < 0.3 was considered small, 0.3–0.5 moderate, and > 0.5 strong.

f EQ value was based on the Swedish value set (Burström K, et al., 2020), ranging from 0.243 to 0.976. Higher values indicate better health.

As hypothesized for the evaluation of convergent validity, EQ LSS correlated strongly with both EQ value (ρ = –0.99 [95% CI: –0.99, –0.98]) and EQ VAS (ρ = –0.59 [95% CI: –0.63, –0.54]). Discriminant validity was also supported as the correlation with life satisfaction was lower for the EQ LSS (ρ = –0.50 [95% CI: –0.55, –0.44]), EQ value (ρ = 0.51 [95% CI: 0.46, 0.56]) and EQ VAS (ρ = 0.58 [95% CI: 0.53, 0.63]) (Table 3).

The sensitivity analysis yielded similar construct validity for the UK and French value sets compared to the Swedish (Table S6).

## Discussion

This is the first study to assess the psychometric properties of EQ-5D-5L amongst OHCA survivors. The study showed that the five health dimensions seem to reflect an underlying latent variable of health status, supporting the use of EQ LSS. The EQ LSS scale showed good internal consistency and negligible DIF for age. The strong association with functional outcome by mRS also supports its construct validity.

There were no clear differences in construct validity of EQ LSS compared with EQ value, which is supported by others.^44^, ^45^ The choice should instead align with the aim of the research, whether survivors’ perspective or health-economic calculations are prioritized. The strength of the EQ LSS is that it is easy to calculate and provides a score based on the patient’s own response instead of being calculated in relation to population norm values.^46^ In multinational trials, determining which national value set to apply can be challenging. Using UK and French value sets yielded similar construct validity results to the Swedish value set, suggesting minimal impact of value set choice in this sample. Although inter-country differences were smaller than clinical differences in OHCA survivors, they may still complicate comparisons between trials.

The most prominent psychometric strength of the EQ LSS was the strong association with functional outcome. This was in line with our expectations and indicates support for its construct validity. This highlights its ability to capture survivors’ health in different functional outcome groups after OHCA and suggests potential utility in research settings. While mRS provides a clinician-reported measure of functional outcome, EQ LSS reflects health from the survivors’ perspective. Although, interrelated, they capture distinct aspects of survivorship, which emphasizes the importance of assessing outcomes after OHCA from several perspectives.^1^

The EQ VAS demonstrated slightly less robust construct validity compared to EQ LSS and EQ value. EQ VAS appeared to capture additional, unknown aspects of health amongst survivors, and showed a stronger association with overall life satisfaction. This raises questions about challenges in measuring complex constructs like health-related quality of life (HRQoL). Despite that EQ-5D-5L often is described as a HRQoL measure,^47^ it might not capture important quality-of-life attributes that matter to individuals.^48^ Consequently, EQ-5D-5L may not reflect OHCA survivors' HRQoL, which underscores the need to distinguish between health status and HRQoL as different constructs.^49^

The strong association between EQ LSS and EQ value was expected but may have been exacerbated by the large proportion reporting best possible health. The observed proportions of survivors with no problems align with findings of other OHCA outcome studies,^50^, ^51^ suggesting this pattern is characteristic of OHCA survivors’ health status. Our results were also comparable with the Swedish population norms, with identical median scores on EQ VAS (80 versus 80).^18^, ^52^

While EQ LSS exhibits several psychometric strengths, important problems remain. Notably, 35% of our sample reported no problems in any dimension, compared with 24% in the reference population,^52^ suggesting greater polarization in health status. Mistargeting, particularly due to this floor effect, raises concerns about the instrument’s ability to measure health status amongst individuals with fewer health problems. This aligns with previous research showing weaker performance of EQ LSS in healthy individuals.^6^ As a result, the EQ-5D-5L may have reduced responsiveness to detect subtle health improvements, particularly amongst those already reporting good health, due to floor effects limiting detectable change. In contrast, prior studies, have demonstrated the relevance and construct validity of EQ-5D-5L across populations with more complex health challenges, such as chronic conditions.^53^, ^54^, ^55^ Moreover, EQ-5D-5L has been criticized for failing to capture key aspects of health, such as fatigue and cognition.^48^ The weaker association with cognitive function suggests that EQ-5D-5L may be less sensitive to cognitive impairments in OHCA survivors. To address these issues, adding items focused on common challenges in OHCA survivors, such as cognition, fatigue, and sleep,^56^, ^57^ could improve construct validity,^58^ targeting and reduce floor effects.^59^ Still, combining generic health measures with more specific tools to enhance granularity is recommended.^1^, ^60^ This also highlights the need for a cardiac arrest-specific survivor-reported outcome measure, an effort that is currently underway.^61^

We identified significant DIF for age, particularly for *Mobility* and *Anxiety/depression*, which is similar to the results of a previous study.^13^ In contrast, the minimal effect sizes found for these DIFs suggest their impact is negligible within this sample. Thus, our findings support that item responses depend on levels of health status, rather than the respondent’s age, enhancing the applicability of EQ LSS across age groups.

## Strengths and limitations

The major strengths of this study are the international setting, large sample size, protocolized follow-up and central monitoring. Accounting for the ordinal item-level data in the analyses is another strength. However, some important limitations need to be addressed.

While the EQ-5D-5L was not developed as an all-reflective scale, it likely includes both reflective and formative indicators.^9^, ^10^ However, given that the use of EQ LSS is based on the assumption of an underlying health construct, our rationale for conducting a CFA for ordinal data was to evaluate this latent structure. This approach was chosen to test whether EQ LSS functions as a unidimensional measure of health status, given its practical application as a potential outcome measure in OHCA research. Our findings indicating a unidimensional latent structure align with previous research in patients with heart failure,^7^ supporting the use of EQ LSS as an outcome measure in this context. While these findings support its use in research settings, further evidence is needed before recommending EQ LSS for routine use in clinical practice.

Our sample may be subject to selection bias, skewed towards survivors with more favourable outcomes. This could be because of the practice of withdrawal of life-sustaining therapy, which has made severe brain injuries uncommon amongst survivors.^57^ This skewed distribution of disability levels could potentially lead to instable effect size estimates, and the magnitude of group differences should be interpretated with caution.

Although proxy ratings can be valuable in capturing outcomes amongst severely impaired survivors, they may overestimate physical limitations and underestimate subjective experiences.^62^ Because the EQ-5D-5L is intended as a self-reported questionnaire, including proxy ratings could introduce measurement bias. To ensure that the psychometric properties reflected survivors’ own perceptions, we excluded proxy ratings to minimize this bias. Future studies should explore methods for integrating proxy ratings to better capture the full spectrum of health outcomes in survivors.

While we acknowledge that the gender imbalance in our sample (84% male) was greater than typically reported in OHCA epidemiology (57–72%),^63^ it was comparable to similar studies (64–94%).^60^ Lower survival rates and poorer neurological prognosis for women can, at least partly, explain this discrepancy.^64^

Another limitation is that we assessed the psychometric properties from only a classical test theory (CTT) perspective.^33^ Future research should address this by employing multiple psychometric techniques, including item response theory framework as conducted in other contexts.^65^ This could offer a robust framework that could be applied to improve the precision of health status measurement in OHCA survivors.

Although 24-months data were collected in the TTM2-trial, they were unavailable for this study, limiting it to a cross-sectional design. Consequently, longitudinal studies are needed to assess the EQ-5D-5L's responsiveness to change over time.

While broader external comparators like SF-36v2 would have strengthened the construct validity, these were not included in the TTM2-trial dataset. This limits the strength of the convergent validity assessment, and we acknowledge this as a limitation in the current analyses.

## Conclusions

The present study found that the psychometric properties of EQ LSS support its use as a measure of health status in OHCA research. The strong association between health and functional dependency indicates robust and comparable construct validity for EQ LSS, EQ value, and EQ VAS in this sample. In the future, the choice of which outcome measure to use should be guided by the aim of the specific study.

## Conflict of interest

The co-author Tobias Cronberg serves as a member of the editorial board of the Resuscitation journal. All other authors declare that they have no known competing financial interests or personal relationships that could have appeared to influence the work reported in this paper.

## Funding sources

This study was supported by the Swedish Heart-Lung Foundation, the Skane Regional University Health-care System Funding, the Skane University Hospital Foundations, the Hans-Gabriel and Alice Trolle-Wachtmeisters Foundation for Medical Research, Grants from the Swedish state under the agreement between the Swedish government and the county councils–the ALF-agreement. Open access funding was provided by Lund University.

The funding bodies had no role in the design of the study; the data collection, analysis, or interpretation; or the writing of the manuscript.

## Availability of data and materials

The data that support the findings of this study are available from the authors upon reasonable request and with permission from the TTM2-trial steering group.

## CRediT authorship contribution statement

**Mattias Bohm:** Conceptualization, Data curation, Formal analysis, Funding acquisition, Investigation, Methodology, Project administration, Visualization, Writing – original draft. **Kristofer Årestedt:** Conceptualization, Formal analysis, Methodology, Supervision, Validation, Visualization, Writing – original draft. **Susann Ullén:** Data curation, Investigation, Methodology, Validation, Visualization, Writing – original draft. **Niklas Nielsen:** Investigation, Resources, Writing – review & editing. **Josef Dankiewicz:** Data curation, Investigation, Resources, Writing – review & editing. **Hans Friberg:** Investigation, Writing – review & editing. **Erik Blennow Nordström:** Data curation, Investigation, Writing – review & editing. **Alain Cariou:** Investigation, Writing – review & editing. **Janus Christian Jakobsen:** Investigation, Writing – review & editing. **Anders Morten Grejs:** Investigation, Writing – review & editing. **Matthias Haenggi:** Investigation, Writing – review & editing. **Naomi E. Hammond:** Investigation, Writing – review & editing. **Katarina Heimburg:** Investigation, Writing – review & editing. **Thomas R. Keeble:** Investigation, Writing – review & editing. **Christoph Leithner:** Investigation, Writing – review & editing. **Christian Rylander:** Investigation, Writing – review & editing. **Johan Undén:** Investigation, Writing – review & editing. **Matt P. Wise:** Investigation, Writing – review & editing. **Tobias Cronberg:** Conceptualization, Funding acquisition, Investigation, Methodology, Project administration, Resources, Supervision, Validation, Visualization, Writing – original draft. **Gisela Lilja:** Conceptualization, Data curation, Funding acquisition, Investigation, Methodology, Project administration, Resources, Supervision, Validation, Visualization, Writing – original draft.

## Ethics approval and consent to participate

Ethical application was approved by the Regional Ethics Committee at Lund University (2015/228). Ethical applications were submitted to all relevant ethics boards and subsequently approved in all participating countries. The trial was conducted in accordance with Good Clinical Practice guidelines and the Helsinki Declaration. All participants provided written informed consent to participate.

## Declaration of competing interest

The authors declare the following financial interests/personal relationships which may be considered as potential competing interests: The co-author Tobias Cronberg serves as a member of the editorial board of the Resuscitation journal. All other authors declare that they have no known competing financial interests or personal relationships that could have appeared to influence the work reported in this paper.
