# Supplementary material for: Psychometric evaluation of EQ-5D-5L in OHCA survivors from the TTM2 trial: a post hoc analysis
Source: Resusc Plus. 2025 May 29;24:100994. doi: 10.1016/j.resplu.2025.100994 (PMC12173684; doi:10.1016/j.resplu.2025.100994)
Supplement: Supplementary Tables [file mmc1.docx]

# Appendix A. Supplementary material

# Psychometric evaluation of EQ-5D-5L in OHCA survivors: a *post hoc* analysis of the TTM2 trial

## Author names

Mattias Bohm^*^, Kristofer Årestedt, Susann Ullén, Niklas Nielsen, Josef Dankiewicz, Hans Friberg, Erik Blennow Nordström, Alain Cariou, Janus Christian Jakobsen, Anders Morten Grejs, Matthias Haenggi, Naomi E Hammond, Katarina Heimburg, Thomas R Keeble, Christoph Leithner, Christian Rylander, Johan Undén, Matt P Wise, Tobias Cronberg, Gisela Lilja.

^*^ Corresponding author:

Mattias Bohm (mattias.bohm@med.lu.se), Department of Intensive and Perioperative Care, Skåne University Hospital Malmö, Inga Marie Nilssons gata 47 F, SE-20502 Malmö, Sweden.

## Table of contents

Table S1. Detailed description of evaluated psychometric properties 3

Table S2. Frequency and percentage of respondents by country 6

Table S3. Descriptive statistics of individual EQ-5D-5L dimension responses 7

Table S4. Internal consistency, precision, and targeting of EQ LSS 8

Table S5. Evaluation of differential item functioning (DIF) 9

Table S6. Sensitivity analysis of EQ value 10

| Table S1. Detailed description of each evaluated psychometric properties analyses of the EQ-5D-5L, their criteria and interpretation. | | |
| --- | --- | --- |
| Psychometric property | Statistical evaluation | Criteria and interpretation |
| Latent structure | Confirmatory Factor Analysis (CFA)  Model statistics, χ^2^  Goodness-of-model fit:  Comparative Fit Index (CFI) ≥ 0.95  Tucker-Lewis Index (TLI) ≥ 0.95  Root Mean Square Error of Approximation (RMSEA) ≤ 0.06  Standardized Root Mean Square Residual (SRMR) ≤ 0.08 | The CFA was based on polychoric correlations with weighted least square mean and variance (WLSMV) adjusted estimation to account for the ordinal item-level data (Brown, 2015). Both the variance of the latent factor as well as the factor loadings are standardized. The χ^2^ statistics of the model was expected to be non-significant and acceptable model fit indices: CFI; TLI; RMSEA; and SRMR (Hu & Bentler, 1999), to indicate support of the hypothesised unidimensional latent structure of the EQ-5D-5L. |
| Differential Item Functioning (DIF) for age | Multiple Indicators, Multiple Causes (MIMIC) modelling in two steps:  1. Baseline model regressing the latent factor on the covariate age to capture group mean differences.  2. Five separate nested models adding direct regression paths from age to each indicator. Each model was compared with the baseline model using a χ^2^ difference test with Satorra-Bentler’s scaling corrections for the WLSMV estimation procedure (Satorra & Bentler, 2001). | The MIMIC analyses were based on the CFA model. Associations between the latent variable, the items and the covariate age are reported with standardized regression coefficients. Positive regression coefficients indicate worse health with increasing age. Magnitude of difference between models was assessed with change in *R²* (Δ*R^2^*) as effect size. With a rejection of the null hypothesis, Δ*R^2^* < 0.02 indicates negligible DIF, 0.02 ≤ Δ*R^2^* < 0.13 indicates small DIF, 0.13 ≤ Δ*R^2^* ≤ 0.26 indicates moderate DIF, and Δ*R^2^* > 0.26 indicates a large DIF effect (Cohen, 1992). |
|  | | |

| Table S1 (continued). Detailed description of each evaluated psychometric properties analyses of the EQ-5D-5L, their criteria and interpretation. | | |
| --- | --- | --- |
| Psychometric property | Statistical evaluation | Criteria and interpretation |
| Internal consistency and precision |  | Analyses specifically of the EQ level sum score (EQ LSS). |
| Internal consistency | Polychoric-based ordinal version of coefficient alpha (α) | As indication of the scale’s reliability, internal consistency was assessed with ordinal alpha, to account for the ordinal nature of the data (Gadermann et al., 2012). Alpha coefficients > 0.7 were acceptable. Traditional coefficient alpha was also calculated for comparative reasons. |
|  | Ordinal omega (ω) | Ordinal omega based on the CFA model was also used to estimate internal consistency. |
| Precision | Standard error of measurement (SEM):  $\sigma\times\sqrt{1-\alpha}$; where *σ* is the standard deviation (SD) and *α* is the internal consistency coefficient of the ordinal alpha. | SEM was calculated as an estimation of score precision (Hobart & Cano, 2009), and was expected to be less than half of the total score’s SD. |
| Targeting | Score distribution | Average score should be near midpoint of scale and cover its potential range. Targeting indicates how the sample’s score aligns with the range of the scale. |
|  | Floor / ceiling effects | Floor / ceiling effects, i.e., proportion of respondents with the lowest or highest possible score, were expected to be < 15% (Terwee et al., 2007). |
|  | Percentage of possible health states utilized | Number and percentage of different health states that were utilized in this sample out of all possible health states (5^5^ = 3125). |

| Table S1 (continued). Detailed description of each evaluated psychometric properties analyses of the EQ-5D-5L, their criteria and interpretation. | | |
| --- | --- | --- |
| Psychometric property | Statistical evaluation | Criteria and interpretation |
| Construct validity |  | Same analyses of construct validity were conducted for all three EQ‑5D‑5L outcome measures: EQ LSS, EQ value [Swedish value set], and EQ VAS. Effect sizes for the three outcomes were compared on each test. The EQ value was based on the national value set from the country with most respondents with complete EQ-5D-5L data in the current study. The Swedish (Burström K, et al., 2020) value set based on an experienced-based time trade-off evaluation method (model 5). |
|  | Sensitivity analysis | To assess if value sets from other countries impact the construct validity of EQ value, the UK (Hernández Alava M, et al, 2023) and French (Andrade L F, et al., 2020) value sets were used. The UK value set is age and sex adjusted. |
| Hypotheses testing |  | Comparing known-groups and determining the ability to differentiate between groups expected to vary. |
| Modified Rankin Scale (mRS) | Kruskal-Wallis test with eta squared ($\eta_{H}^{2})$, as effect size estimate | Effect size ($\eta_{H}^{2}$): < 0.06 (small), 0.06–0.14 (moderate), and > 0.14 (large) (Tomczak & Tomczak-Łukaszewska, 2014). Survivors with higher mRS scores were expected to report more health problems, with at least a small effect size (Golicki et al., 2015). |
| Montreal Cognitive Assessment (MoCA) | Mann-Whitney U test with $r$, as effect size estimate | Survivors with cognitive problems (MoCA < 26) were expected to report more health problems than those without cognitive problems (≥ 26) with at least a small effect size (Keetharuth et al., 2022). Effect size (*r)*: < 0.3 (small), 0.3–0.5 (moderate), and > 0.5 (large) (Cohen, 1988). |
| Convergent validity | Spearman correlations (ρ) | Comparison of measures that measure similar concept. Correlations between EQ LSS, EQ value, and EQ VAS were expected to be strong. Effect size for coefficient ρ: < 0.3 (small), 0.3–0.5 (moderate), and > 0.5 (strong) (Cohen, 1988). |
| Discriminant validity | Spearman correlations (ρ) | Comparison of measures that are expected to measure distinct and different concepts and correlate to a lesser degree than convergent measures. Correlations between life satisfaction [single-item, 1–10] and EQ LSS, EQ value, and EQ VAS. Coefficient ρ: < 0.3 (small), 0.3–0.5 (moderate), and > 0.5 (strong) (Cohen, 1988). |
|  | | |

| Table S2. Frequency and percentage of respondents with complete EQ-5D-5L data by country in the TTM2-trial (n = 783). | |
| --- | --- |
| Country, n (%) ^a^ |  |
| Sweden | 162 (21) |
| United Kingdom | 141 (18) |
| Switzerland ^b^ | 123 (16) |
| France | 69 (9) |
| Czech Republic | 67 (9) |
| Norway | 67 (8) |
| Australia | 38 (5) |
| Denmark | 36 (5) |
| New Zealand | 35 (4) |
| Italy | 21 (3) |
| Germany | 20 (3) |
| Belgium | 2 (< 1) |
| Austria | 1 (< 1) |
| Abbreviations: TTM2-trial – Targeted Hypothermia versus Targeted Normothermia after Out-of-Hospital Cardiac Arrest trial.  ^a^ Countries are sorted from highest to lowest number of respondents.  ^b^ Currently no available national value set. | |

| Table S3. Descriptive statistics of individual EQ-5D-5L dimensions among the OHCA survivors from the TTM2-trial (n = 783). | | | | | | |
| --- | --- | --- | --- | --- | --- | --- |
|  |  | Levels of perceived problems (abridged) ^a^ | | | | |
|  | Mdn (Q1–Q3) | No problems,  n (%) | Slight problems,  n (%) | Moderate problems,  n (%) | Severe problems,  n (%) | Extreme problems,  n (%) |
| Mobility | 1 (1–2) | 572 (73) | 114 (15) | 72 (9) | 20 (3) | 5 (1) |
| Self-care | 1 (1–1) | 695 (89) | 62 (8) | 17 (2) | 5 (1) | 4 (1) |
| Usual activities | 1 (1–2) | 536 (68) | 141 (18) | 74 (9) | 18 (2) | 14 (2) |
| Pain/discomfort | 1 (1–2) | 446 (57) | 196 (25) | 112 (14) | 26 (3) | 3 (< 1) |
| Anxiety/depression | 1 (1–2) | 480 (61) | 197 (25) | 82 (10) | 19 (2) | 5 (1) |
| Abbreviations: Mdn – median; OHCA – out-of-hospital cardiac arrest; Q1–Q3 – quartile 1 to 3; TTM2-trial – Targeted Hypothermia versus Targeted Normothermia after Out-of-Hospital Cardiac Arrest trial.  ^a^ No problems coded as ‘1’; Slight problems coded as ‘2’; Moderate problems coded as ‘3’; Severe problems coded as ‘4’; Extreme problems coded as ‘5’. | | | | | | |

| Table S4. Internal consistency reliability, precision, and targeting of the EQ-5D-5L level sum score (EQ LSS) among OHCA survivors at 6 months from the TTM2-trial (n = 783). | |
| --- | --- |
| Psychometric property |  |
| Internal consistency reliability |  |
| Ordinal α ^a^ | 0.88 |
| Ordinal α when item deleted, min–max ^b^ | 0.83–0.89 |
| Coefficient α ^a^ | 0.79 |
| Coefficient α when item deleted, min–max ^b^ | 0.72–0.79 |
| Ordinal omega (ω) ^c^ | 0.81 |
| Precision |  |
| SEM, ordinal α based ^d^ | 1.01 |
| Targeting |  |
| Possible score range | 5–25 |
| Mean (SD) score ^e^ | 7.3 (2.9) |
| Min–max score ^f^ | 5–25 |
| Floor effect, n (%) ^g^ | 274 (35) |
| Ceiling effect, n (%) ^h^ | 1 (< 1) |
| Skewness ^i^ | 1.8 |
| Different health states utilized, n (%) ^j^ | 178 (6) |
| Abbreviations: CI – Confidence Interval; OHCA – out-of-hospital cardiac arrest; SD – Standard Deviation; SEM – Standard Error of Measurement; TTM2-trial – Targeted Hypothermia versus Targeted Normothermia after Out-of-Hospital Cardiac Arrest trial.  ^a^ Should be ≥ 0.80.  ^b^ Should not increase compared with α for the total score.  ^c^ Ordinal omega was based on the revised confirmatory factor analysis model.  ^d^ Should be less than half of the total score SD.  ^e^ Should be close to scale midpoint.  ^f^ Should span most of the scale’s score range.  ^g^ Indicate no problems in any dimension. Should be < 15%.  ^h^ Indicate extreme problems in all dimensions. Should be < 15%.  ^i^ Should be between −1 and +1.  ^j^ Number and percentage of different health states utilized out of all possible health states (5^5^ = 3125). | |

| Table S5. Evaluation of DIF for age in EQ-5D-5L based on MIMIC CFA models among the OHCA survivors from the TTM2-trial (n = 783). | | | | | | |
| --- | --- | --- | --- | --- | --- | --- |
|  |  | DIF models EQ-5D-5L items ^b^ | | | | |
|  | No-DIF model ^a^ | Mobility | Self-care | Usual activities | Pain/discomfort | Anxiety/depression |
| Direct effects - Factors ^c^ |  |  |  |  |  |  |
| Latent variable regressed on age | 0.099* | 0.027 | 0.089* | 0.120** | 0.098* | 0.161*** |
| Direct effects - Items ^c^ |  |  |  |  |  |  |
| Item regressed on age | n/a | 0.293*** | 0.061 | −0.065* | 0.004 | −0.237*** |
| Chi-square difference test ^d^ |  |  |  |  |  |  |
| χ^2^ (df) | n/a | 57.6 (1) | 1.4 (1) | 4.1 (1) | 0.01 (1) | 36.5 (1) |
| p-value | n/a | < 0.001 | 0.233 | 0.044 | 0.921 | < 0.001 |
| *R^2^* values ^e^ |  |  |  |  |  |  |
| No-DIF model | n/a | 0.698 | 0.799 | 0.722 | 0.524 | 0.398 |
| DIF model | n/a | 0.717 | 0.800 | 0.722 | 0.524 | 0.415 |
| Δ*R^2^* (DIF – No-DIF) | n/a | 0.019 | 0.001 | < 0.001 | < 0.001 | 0.017 |
| Abbreviations: CFA – Confirmatory Factor Analysis; df – degrees of freedom; DIF – differential item functioning; MIMIC – multiple indicators, multiple causes; n/a – not applicable; OHCA – out-of-hospital cardiac arrest; TTM2-trial – Targeted Hypothermia versus Targeted Normothermia after Out-of-Hospital Cardiac Arrest trial.  * p < 0.05; ** p < 0.01; *** p < 0.001.  ^a^ Only the latent variable is regressed on age, as baseline model.  ^b^ Both the latent variable and item are regressed on age.  ^c^ Values are reported as standardized regression coefficients.  ^d^ Scaled χ^2^ difference test (Satorra-Bentler’s scaling corrections) between the DIF model and No-DIF model, adjusted for the weighted least square mean and variance estimation procedure of the CFA.  ^e^ Explained variance in each item for the No-DIF model, DIF model, and the difference in *R^2^* (Δ*R^2^*) between the DIF and No-DIF model. If significant χ^2^ difference test, Δ*R^2^* < 0.02 indicates negligible DIF, 0.02 ≤ Δ*R*^2^ < 0.13 indicates small DIF, 0.13 ≤ Δ*R*^2^ ≤ 0.26 indicates moderate DIF, and Δ*R*^2^ > 0.26 indicates a large DIF effect. | | | | | | |

| Table S6. Sensitivity analysis for construct validity of EQ-5D-5L, amongst OHCA survivors at 6 months from the TTM2-trial (n = 783). | | |
| --- | --- | --- |
| Property | EQ value  [UK value set] ^a^ | EQ value  [French value set] ^b^ |
| **Hypotheses testing** |  |  |
| modified Rankin Scale (mRS) |  |  |
| 0 [n = 281], Mdn (Q1–Q3) | 0.99 (0.89–0.99) | 1.00 (0.98–1.00) |
| 1 [n = 164], Mdn (Q1–Q3) | 0.87 (0.75–0.99) | 0.98 (0.92–1.00) |
| 2 [n = 250], Mdn (Q1–Q3) | 0.80 (0.69–0.91) | 0.95 (0.88–0.98) |
| 3 [n = 58], Mdn (Q1–Q3) | 0.61 (0.51–0.75) | 0.86 (0.75–0.91) |
| 4 [n = 28], Mdn (Q1–Q3) | 0.26 (0.18–0.52) | 0.56 (0.40–0.69) |
| 5 [n = 2], Mdn (Q1–Q3) | –0.29 (–0.41 to –0.17) | –0.20 (–0.36 to –0.04) |
| p-value ^c^ | < 0.001 | < 0.001 |
| Effect size ($\eta_{H}^{2}$) ^d^ | 0.31 | 0.35 |
| Montreal Cognitive Assessment (MoCA) |  |  |
| No cognitive problems [MoCA ≥ 26], Mdn (Q1–Q3) | 0.89 (0.79–0.99) | 0.98 (0.94–1.00) |
| Cognitive problems [MoCA < 26], Mdn (Q1–Q3) | 0.81 (0.64–0.99) | 0.95 (0.86–1.00) |
| p-value ^e^ | < 0.001 | < 0.001 |
| Effect size (*r*) ^f^ | 0.16 | 0.20 |
| **Convergent validity ^g^** |  |  |
| EQ LSS | –0.97 [–0.97, –0.96] | –0.99 [–0.99, –0.99] |
| EQ value [Swedish value set] | 0.94 [0.93, 0.95] | 0.98 [0.98, 0.98] |
| EQ VAS | 0.56 [0.51, 0.61] | 0.59 [0.54, 0.63] |
| **Discriminant validity ^g^** |  |  |
| Life satisfaction | 0.49 [0.43, 0.54] | 0.49 [0.44, 0.54] |
| Abbreviations: EQ LSS – EQ-5D-5L level sum score; Mdn – median; OHCA – out-of-hospital cardiac arrest; TTM2-trial – Targeted Hypothermia versus Targeted Normothermia after Out-of-Hospital Cardiac Arrest trial.  ^a^ UK value set (Hernández Alava M, et al., 2023), ranges from −0.532 to 0.989 (age adjusted for men) and from −0.529 to 0.988 (age adjusted for women). Higher values indicate better health.  ^b^ French value set (Andrade L F, et al., 2020), ranges from −0.525 to 1. Higher values indicate better health.  ^c^ Kruskal-Wallis test.  ^d^ Eta squared ($\eta_{H}^{2}$) effect size: < 0.06 (small effect), 0.06–0.14 (moderate effect), and > 0.14 (large effect).  ^e^ Mann-Whitney U test.  ^f^ Rank-biserial correlation coefficient (r) effect size: < 0.3 (small effect), 0.3–0.5 (moderate effect), and > 0.5 (large effect).  ^g^ Spearman’s rho. Values in square brackets indicate the 95% confidence interval. A coefficient of ρ < 0.3 was considered small, 0.3–0.5 moderate, and > 0.5 strong. | | |
